# mActive-Smoke: A Prospective Observational Study Using Mobile Health Tools to Assess the Association of Physical Activity With Smoking Urges

**DOI:** 10.2196/mhealth.9292

**Published:** 2018-05-11

**Authors:** Luke G Silverman-Lloyd, Sina Kianoush, Michael J Blaha, Alyse B Sabina, Garth N Graham, Seth S Martin

**Affiliations:** ^1^ Ciccarone Center for the Prevention of Cardiovascular Disease Division of Cardiology, Department of Medicine Johns Hopkins University School of Medicine Baltimore, MD United States; ^2^ Department of Epidemiology Johns Hopkins Bloomberg School of Public Health Baltimore, MD United States; ^3^ Aetna Foundation Hartford, CT United States

**Keywords:** activity trackers, cigarette smoking, exercise, fitness trackers, mobile health, mHealth, physical activity, smartphone, smoking, text messaging, texting

## Abstract

**Background:**

Evidence that physical activity can curb smoking urges is limited in scope to acute effects and largely reliant on retrospective self-reported measures. Mobile health technologies offer novel mechanisms for capturing real-time data of behaviors in the natural environment.

**Objective:**

This study aimed to explore this in a real-world longitudinal setting by leveraging mobile health tools to assess the association between objectively measured physical activity and concurrent smoking urges in a 12-week prospective observational study.

**Methods:**

We enrolled 60 active smokers (≥3 cigarettes per day) and recorded baseline demographics, physical activity, and smoking behaviors using a Web-based questionnaire. Step counts were measured continuously using the Fitbit Charge HR. Participants reported instantaneous smoking urges via text message using a Likert scale ranging from 1 to 9. On study completion, participants reported follow-up smoking behaviors in an online exit survey.

**Results:**

A total of 53 participants (aged 40 [SD 12] years, 57% [30/53] women, 49% [26/53] nonwhite) recorded at least 6 weeks of data and were thus included in the analysis. We recorded 15,365 urge messages throughout the study, with a mean of 290 (SD 62) messages per participant. Mean urge over the course of the study was positively associated with daily cigarette consumption at follow-up (Pearson r=.33; *P*=.02). No association existed between daily steps and mean daily urge (beta=−6.95×10^−3^ per 1000 steps; *P*=.30). Regression models of acute effects, however, did reveal modest inverse associations between steps within 30-, 60-, and 120-min time windows of a reported urge (beta=−.0191 per 100 steps, *P*<.001). Moreover, 6 individuals (approximately 10% of the study population) exhibited a stronger and consistent inverse association between steps and urge at both the day level (mean individualized beta=−.153 per 1000 steps) and 30-min level (mean individualized beta=−1.66 per 1000 steps).

**Conclusions:**

Although there was no association between objectively measured daily physical activity and concurrently self-reported smoking urges, there was a modest inverse relationship between recent step counts (30-120 min) and urge. Approximately 10% of the individuals appeared to have a stronger and consistent inverse association between physical activity and urge, a provocative finding warranting further study.

## Introduction

Smoking is the leading cause of preventable death in the world [1]. Although public health campaigns, antismoking laws, and new pharmacotherapies have successfully reduced smoking rates [2], further progress has proven difficult due, in part, to the complex psychosocial nature of tobacco addiction [3]. Although many smokers wish to quit smoking because of knowledge of its harmful effects [4], self-quitting initiatives have shown largely unsuccessful outcomes [5], suggesting that interventions are necessary to assist smokers in cessation. To that end, counseling and pharmacotherapy (ie, nicotine replacement, bupropion, and varenicline) are established smoking cessation interventions, but not effective in most individuals. Although nicotine replacement therapies have been shown to boost smoking cessation efforts twofold compared with placebo [6], 70% to 80% of smokers who use these therapies relapse [7].

Physical activity (PA) has been proposed as an aid for smoking cessation [8] and as a means for harm reduction among smokers who do not wish to quit [9]. A 2014 review that examined 20 trials assessing exercise as an aid for smoking cessation found the evidence for such a recommendation to be insufficient [10]. However, this review presents evidence that exercise may be an effective means for reducing tobacco cravings among smokers who are not presently motivated to quit [10], thereby suggesting exercise as a mediator of harm reduction. A 2014 pilot randomized trial—Exercise Assisted Reduction then Stop (EARS)—found that PA coupled with support for smoking reduction was effective in promoting reduction and cessation among smokers who did not wish to quit immediately [11]. A subsequent study examining data from the EARS trial provided further evidence for the role of PA on smoking reduction but did not find that this association was related to an increase in PA [9]. Rather, evidence suggests that the act of self-monitoring PA [12] and smoking behaviors may improve self-regulation [13] and thus decrease smoking [14] and likelihood of relapse [15] by reinforcing the notion of PA as an aid for smoking reduction [11].

Previous interventional studies have suggested that acute exercise decreases smoking urges [16-22], with activities of medium-long duration and moderate-vigorous intensity displaying the most substantial effects [18]. A systematic review and meta-analysis of individual-level data from 17 trials reported that PA acutely reduces cigarette craving [23]. Using a 2-stage independent participant data meta-analysis, this study assessed the effects of PA on desire to smoke as measured by a 7-point Likert scale and found an average standardized mean difference (SMD) of −2.03 (95% CI −2.60 to −1.46) between PA and control conditions [23]. However, these studies may be limited in clinical applicability because of experimental design and scope of measurement. Regarding the former, most acute studies have involved moderate to heavy smokers, where smoking urges were manipulated by periods of imposed smoking abstinence. These measures were taken to reduce the likelihood of a flooring effect: if participants had been allowed to smoke before these experimental trials, cravings and withdrawal symptoms might have been reduced to none, allowing for no further reduction as a result of exercise. Despite this important consideration, these experimental conditions were not reflective of the everyday circumstances that contribute to the complex manifestation of smoking urges in the natural environment.

Regarding the scope of measurement, these studies only assessed acute effects of exercise on smoking urges, leaving uncertainty around the longitudinal association. Several studies exploring longer-term associations between PA and smoking behaviors [24-30] showed that exercise improved follow-up abstinence rates [24,26,27], increased time until next cigarette [30], led to reductions in smoking and cravings [25,29], and was associated with lower smoking intensity and reduced likelihood of smoking [28]. However, both smoking behaviors and PA measures in these studies were primarily obtained through self-assessment and recall, thus rendering these data susceptible to bias [31,32].

Further studies with improved methodological rigor are needed to address the aforementioned limitations in both acute and longitudinal analyses of PA and smoking behavior [23]. To that end, mobile health (mHealth) technologies have the potential to allow continuous, accurate, and patient-friendly monitoring of health data, including subjective and objective behavioral information [33]. These technologies are especially practical for ecological momentary assessment (EMA) research, designed to sample subjects’ real-time behaviors and experiences in the natural environment and minimize recall bias [34]. Importantly, use of mHealth also brings the potential to address health equity, given the rapidly increasing use of mHealth technologies in low-income individuals [35]. Several studies have validated the utility of activity trackers [36] and text messaging [37] for the measurement of PA and smoking urges, respectively. These technologies have also proven successful in behavioral interventions: leveraging activity tracking and text messaging to promote increased PA [38] or using personalized text messages to enhance smoking cessation [39]. Using both these mHealth tools in the natural environment of individuals who are active smokers, we aimed to assess the real-time association between objectively measured PA and concurrently reported smoking urges by examining the relationship between daily steps and mean daily urges. Secondarily, we sought to examine the acute associations of steps and urges in varying short-term time windows and to assess changes in smoking and PA behaviors over time in association with self-monitoring.

## Methods

### Study Design

For this longitudinal study spanning 12 weeks, the Fitbit Charge HR—a wrist-worn triaxial digital accelerometer with a built-in optical heart rate (HR) monitor that allows for continuous monitoring of activity throughout the day—and smartphone-based short message service (SMS) text messaging were used for data collection. SMS text messaging was also used for participant monitoring; after a face-to-face enrollment visit with the study coordinator, communication occurred via text messages to answer questions, troubleshoot, and send reminders to address nonadherence with the study protocol. Participants who had gaps in their data (ie, missing days of data capture) by the end of 12 weeks were asked if they would voluntarily prolong the duration of their participation to ensure complete data capture.

### Recruitment

We recruited 60 participants from April 7 to September 2, 2016, using several modalities, including on-site advertisements, social media, and physician referral. Before enrollment, participants were screened for eligibility via email and met in-person with a study coordinator to review the consent form and study information. To satisfy inclusion criteria, participants were required to be aged 18 years or older, smoke at least 3 cigarettes per day on average, and own a smartphone. Participants were excluded from enrollment if they were prohibited from normal PA for previously diagnosed health reasons. Face-to-face visits were not required after enrollment.

### Baseline Data Collection

During the in-person meeting with the study coordinator, participants completed an online enrollment questionnaire to record baseline demographic characteristics, PA, and smoking behavior. Baseline PA was assessed using the short form of the International Physical Activity Questionnaire (IPAQ)—a 9-item questionnaire assessing time spent walking, in vigorous- and moderate-intensity activity, and in sedentary activity over a 7-day period [40]. Baseline smoking behavior was obtained using the Arizona Smoking Assessment Questionnaire (ASAQ)—a 27-item questionnaire that assesses past and present tobacco use by recording different types of exposure, quit attempts, and age of smoking onset [41]. In a sample of 600 participants, the ASAQ showed that the number of daily cigarettes and portion of cigarette smoked were significantly predictive of plasma cotinine levels (*P*<.001) [41].

### Measurement and Monitoring of Physical Activity

PA was measured in steps over at least 6 weeks using the Fitbit Charge HR. During the in-person interview with the study coordinator, participants were guided through on-screen instructions for setup. The study coordinator emphasized that participants were expected to wear the device throughout the day, including during exercise; device removal was only advised for swimming or showering, and wearing it to sleep was optional.

Data flowed from the Fitbit device through the smartphone to encrypted Fitbit servers, which stored minute-level data up to 7 days and day-level data for up to 30 days. Fitbit data for all study participants were compiled in Fitabase—a research platform that collects real-time data from activity-tracking devices and stores it in high-security data centers. Fitabase has been used in more than 200 studies as a data analytics platform for Fitbit devices [42]. Fitabase stores data at various levels of granularity—from second-level to day-level—and allows for data export in individualized or batch formats. At the conclusion of the data collection phase, all participant data were downloaded from the Fitabase server and exported as comma separated values (CSV) files, where it was organized and uploaded into Stata (version 14.2; StataCorp, College Station, TX, USA) for analysis.

Fitabase’s live device monitoring feature also allowed for monitoring participants in real-time by reporting syncing activity, battery life, and activity data on a single project dashboard. With access to real-time surveillance, investigators were able to monitor nonadherence, such as delayed syncing activity or failure to wear the device, which was addressed through a series of reminder text messages, emails, and phone calls.

### Measurement of Smoking Urges

Time-stamped smoking urges were quantified via SMS text messages sent by participants. Recurring automatic text message reminders were scheduled for delivery 3 to 4 times per day using an automated messaging service (Boomerang, Baydin Inc, Mountain View, CA, USA). Prompting was used to promote study engagement—an evidence-based strategy that has been shown to improve participant engagement with digital interventions compared with no strategy [43]. Timing of text messages was customized based on participant preference. Each text contained an identical message, asking participants to express their instantaneous urge for smoking on a 9-point Likert scale—a previously validated measure of self-reported craving [44,45] consistent with EMA data sampling methods [34]. Low urge was indicated by a value of 1. Participants were permitted and encouraged to send as many text messages as possible, with or without prompting, but were asked to send a minimum of 3 messages per day. Participants also received weekly recurring messages that included brief instructions and reminders to sync their Fitbit devices. Additional reminder messages were scheduled for participants who displayed consistent patterns of nonadherence.

### Follow-Up Data Collection

At the conclusion of the study, participants were asked to complete an online exit survey to provide their perceptions about the associations between PA and smoking urges and behavior. The IPAQ-short form and ASAQ were readministered in the exit survey, allowing for a comparison of these measures between baseline and follow-up.

### Statistical Analyses

We estimated that a sample size of 50 participants each contributing at least 20 days of complete data capture and accounting for intraindividual correlation of the repeated data measurement would yield 90% power to detect a correlation of .12 between steps per day and mean reported smoking urges per day.

Baseline characteristics were summarized using descriptive statistics—frequency (percentage) for categorical data, and mean (SD) and median (interquartile range) for continuous data. Follow-up data were summarized in an identical manner and compared with baseline characteristics for variables of interest. Trends in mean daily urge and daily steps between baseline and follow-up were also examined categorically. To do so, a midpoint was calculated for each participant, which represented the day at which study participation was 50% complete. Mean daily urge and mean daily steps were calculated before and after this midpoint for each participant. This categorization allowed for broad changes in these 2 measures to be assessed between the first and second halves of the study. Furthermore, this grouping allowed for mean daily steps and mean daily urge to be included in comparisons of other baseline and follow-up measures, such as self-reported PA and smoking behavior. Outliers were defined as values 1.5 times the interquartile range above the upper quartile or below the lower quartile.

A series of protocols were developed to distinguish between wear and nonwear time. Because participants were not required to wear their devices to sleep, we targeted the time window of 10:00 AM to 10:00 PM in determining wear time. We determined HR data to be the most reliable predictor of wear time, as the Fitbit Charge HR is designed to record HR data at 1-s intervals during exercise and at 5-s intervals all other times [46]. Thus, we interpreted the presence of HR data as evidence of wear time. Drawing from prior literature on determining wear time criteria [47], we defined nonwear time as 90 consecutive minutes of missing HR data. Days that included 2 or more of these 90-min consecutive nonwear windows were excluded from day-level analysis. These criteria were implemented to avoid imposing arbitrary cutoffs on our determination of data validity. Days in which total wear time was less than 6 hours within our target time window were also excluded. At least 6 total weeks of recorded data were required for participants to be included in the analysis.

Change in daily cigarette consumption was defined as the difference between the number of cigarettes smoked per day at baseline and follow-up as reported by participants in the enrollment and exit surveys, respectively. In addition, linear regression models were run to assess the change in daily steps and daily urge over time, as measured by the beta coefficients in the regression models. To assess the acute effects of PA, prespecified analyses were performed to assess minute-level associations between steps and urge within 5-, 30-, 60-, and 120-min time windows before urge reporting. These time windows were informed by results from prior studies, which found that acute bouts of low-intensity exercise reduced smoking urges for anywhere from 20 min [48] to 50 min [30] postexercise. The 120-min time window was included to assess whether and to what extent this effect might be prolonged after an acute bout of PA.

Structurally, the data contained both longitudinal and cross-sectional dimensions and can thus be best classified as panel data [49]. Feasible generalized least squares (FGLS) regression models were used to analyze the relationship between mean urge per day (dependent variable) and daily steps (independent variable). This procedure is recommended for panel data that are unbalanced or unequally spaced [50], and appropriate when the number of time points (T) exceeds the number of cross-sections (N) [51]—both of which are characteristic of our data. Furthermore, this model allowed us to correct for heteroscedasticity—unequal variance of the dependent variable across a range of values of an independent variable—which can lead to inefficient parameter estimates and faulty inferences [52].

Analyses were performed to explore interactions by age, sex, baseline PA and smoking levels, race/ethnicity, and intention to quit during the study period. For these analyses, binary definitions of demographic variables were used based on the following cutoffs: age ≥40 years, cigarettes per day ≥10 for baseline smoking level, high activity or not for IPAQ-measured baseline PA, white/nonwhite for race/ethnicity, and yes/no for intention to quit during the study period. Given the sample size, a *P* value of .10 or less was considered evidence of interaction. Furthermore, exploratory subgroup analyses were performed to examine heterogeneity in individuals, allowing for the identification of certain participants that showed a consistently strong association between steps and urge. This approach was used in an effort to account for individual variability in outcomes, consistent with the focus of the precision medicine initiative (PMI) [53]. To explore heterogeneity in individuals, linear regression models were run to calculate the association between daily steps and mean daily urge for each participant. Point estimates and CIs were analyzed for each participant, allowing us to determine which particular individuals exhibited strong associations between daily steps and mean daily urge.

## Results

### Participant and Data Flow

**Figure 1:**
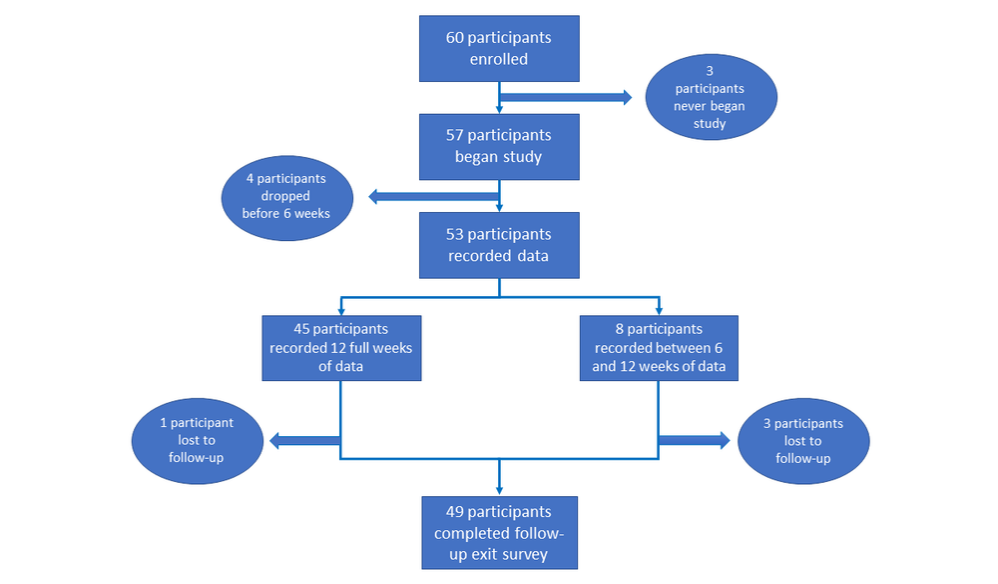
Participant flow.

The study flow diagram is shown in Figure 1. A total of 53 participants recorded data for at least 6 weeks and were thus included in the analysis. Of 53 participants, 49 participants completed the online exit survey at the conclusion of the study, and 4 participants were lost to follow-up.

### Baseline Characteristics

Mean age was 40 (SD 12) years, with 57% (30/53) women and 49% (26/53) nonwhite participants. Moreover, 30% (16/53) had a Bachelor’s degree or higher and 38% (20/53) were obese, whereas 53% (28/53) were in the high activity category as defined by IPAQ assessment. Participants smoked 12 (SD 8) cigarettes per day and had been smokers for 19 (SD 12) years (Table 1).

### Data Capture

In total, participants recorded 4445 complete days of data, with a mean of 84 (SD 12) days per participant; 866 days were eliminated from analysis based on nonwear criteria. A total of 3579 of all days (81%) were eligible for analysis after applying exclusion criteria. Participants sent a total of 15,365 urge messages throughout the study, with a mean of 290 (SD 62) messages per participant. The majority (approximately 80%) of urge messages sent by participants were prompted.

Men reported modestly higher mean urges than women: 5.56 (95% CI 5.50-5.62) versus 5.19 (95% CI 5.13-5.24), respectively. Men also recorded significantly higher mean daily steps than women by 2994 (95% CI 2693-3294; *P*<.001).

### Urge Validation

Participants’ mean urge over the course of the study was positively associated with the number of cigarettes smoked per day as reported in the exit survey (Pearson r=.33, *P*=.02). Furthermore, mean urge over the last week of each participant’s study duration was significantly correlated with daily cigarette consumption (r=.37; *P*=.01).

### Association Between Steps and Urge: Day-Level and Acute

**Figure 2:**
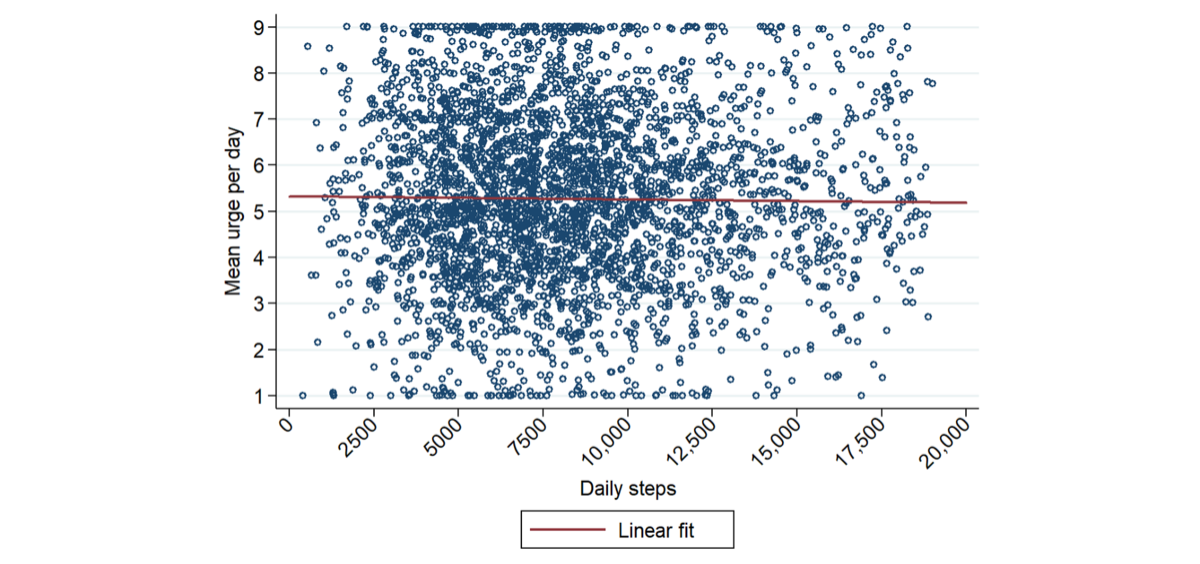
Mean urge per day plotted against daily steps, after applying exclusion criteria and omitting outliers.

Considering the day-level association, as shown in Figure 2, there was a wide range of PA levels and a full representation of urges, without clear relation between them. In formal quantitative assessment, we found no significant association between daily steps and mean daily urge (beta=−6.95×10^−3^ per 1000 steps; *P*=.30). In an adjusted model controlling for age, sex, baseline PA and smoking levels, and race/ethnicity, our primary outcome between daily steps and mean daily urge remained null (beta=−1.18×10^−2^ per 1000 steps; *P*=.11).

Regression models of acute effects, however, did reveal modest inverse associations between steps within 30-, 60-, and 120-min time windows of a reported urge, which were all significant at the *P*<.01 level (Table 2). The strongest association was observed for 30 min of accumulated steps before an urge, with a 0.0191 lower urge per 100 steps accumulated in this time. This translated to an approximately 0.2 lower urge for 1000 steps accumulated over 30 min. The estimate for the effect was reduced by approximately 50% for the 1-hour time frame and another approximately 50% for the 2-hour time frame.

### Exploratory Subgroup Analyses

For the interaction of steps per day with demographic factors, *P* values for interaction were as follows: .73 for age; .10 for sex (significant inverse relationship in men only, beta=−2.65×10^−2^ per 1000 steps, 95% CI −4.36×10^−2^ to −9.27×10^−3^); .45 for race/ethnicity; <.01 for baseline PA (significant inverse relationship in *high-activity* participants, beta=−3.14×10^−2^ per 1000 steps, 95% CI −5.0×10^−2^ to −1.28×10^−2^; significant positive relationship in non-high-activity participants, beta=3.45×10^−2^ per 1000 steps, 95% CI 1.58×10^−2^ to 5.32×10^−2^); .71 for baseline smoking level; and <.01 for intention to quit (significant inverse relationship for *yes* respondents only, beta=−3.5×10^−2^ per 1000 steps, 95% CI −5.6×10^−2^ to −1.5×10^−2^).

**Figure 3:**
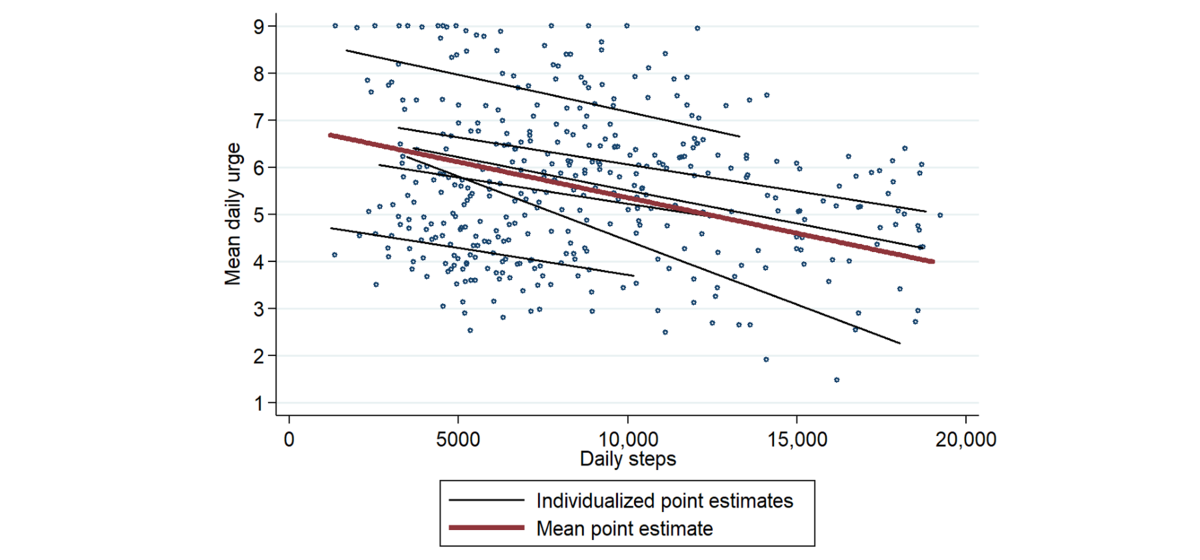
Mean urge per day plotted against daily steps for the 6 “extreme responders,” after applying exclusion criteria and omitting outliers.

**Figure 4:**
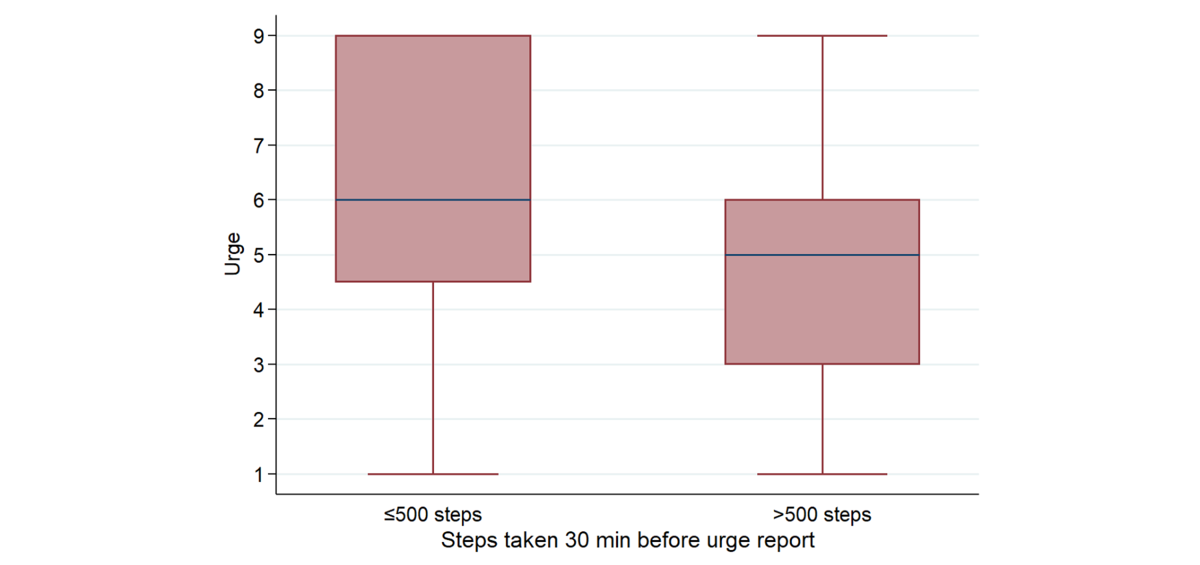
Boxplot of urge for the 6 “extreme responders,” stratified by episodes in which ≤500 or >500 steps were taken in the 30-min time window before an urge report.

One participant exhibited a consistent positive association between steps and urge, whereas a subset of 6 participants (11%) exhibited a consistent inverse relationship between steps and urge. At the day-level, these so-called extreme responders to PA exhibited a mean individualized point estimate of −0.15 decrease in urge per 1000 steps (95% CI −0.22 to −0.09; Figure 3). In the analysis assessing the relationship between steps and urge in the 30-min time window preceding an urge, these individuals showed a mean individualized point estimate of −1.66 decrease in urge per 1000 steps (95% CI −2.48 to −0.84). In addition, data from the 30-min window before an urge for these 6 individuals were stratified around a step cutoff of 500 (Figure 4); episodes in which 500 or fewer steps were taken had a mean urge of 6.22 (95% CI 5.90-6.54), whereas episodes in which more than 500 steps were taken had a mean urge of 4.80 (95% CI 4.55-5.05). Acute inverse associations between steps and urge were largely driven by these 6 individuals, and results became null on excluding them from the analysis.

**Table 1:** Baseline characteristics of mActive-Smoke participants.

Characteristic		mActive-Smoke Participants (N=53)
**Sex, n (%)**		
	Men	23 (43)
	Women	30 (57)
White race, n (%)		27 (51)
Age in years, mean (SD)		40 (12)
Married, n (%)		15 (28)
**Education, n (%)**		
	HS^a^ diploma or less, including general education diploma (GED)	8 (15)
	Associate’s degree/some college credit	29 (55)
	Bachelor’s degree or higher	16 (30)
Employed, n (%)		43 (81)
**BMI^b^ (kg/m^2^), mean (SD)**		29 (6)
	≥30, n (%)	20 (38)
**IPAQ^c^, n (%)**		
	Low	6 (11)
	Moderate	19 (36)
	High	28 (53)
Sedentary hours on a weekday, mean (SD)		6.8 (3.3)
**Cigarettes smoked per day, n (%)**		
	≤10	34 (64)
	>10	19 (36)
Age started smoking, mean (SD)		18 (5)
Years as a smoker, mean (SD)		19 (12)
Pack-years^d^, mean (SD)		14 (12)
**Type of recruitment, n (%)**		
	On-site advertisement	30 (56)
	Social media	20 (38)
	Physician referral	3 (6)

^a^HS: high school.

^b^BMI: body mass index.

^c^Categories defined by International Physical Activity Questionnaire (IPAQ) guidelines.

^d^Defined as (mean cigarettes per day/20) × number of years as a smoker.

**Table 2:** Feasible generalized least squares regression results of smoking urge versus steps over various time windows before urge reports.

Steps accumulated within various time windows of urge reporting	Association of urge with steps (beta coefficient, per 100 steps)	*P* value	95% CI (per 100 steps)
30 min before	−0.0191	<.001	−0.0284 to −0.0098
60 min before	−0.00891	.003	−0.0147 to −0.0031
120 min before	−0.00495	.007	−0.00851 to −0.00138

**Figure 5:**
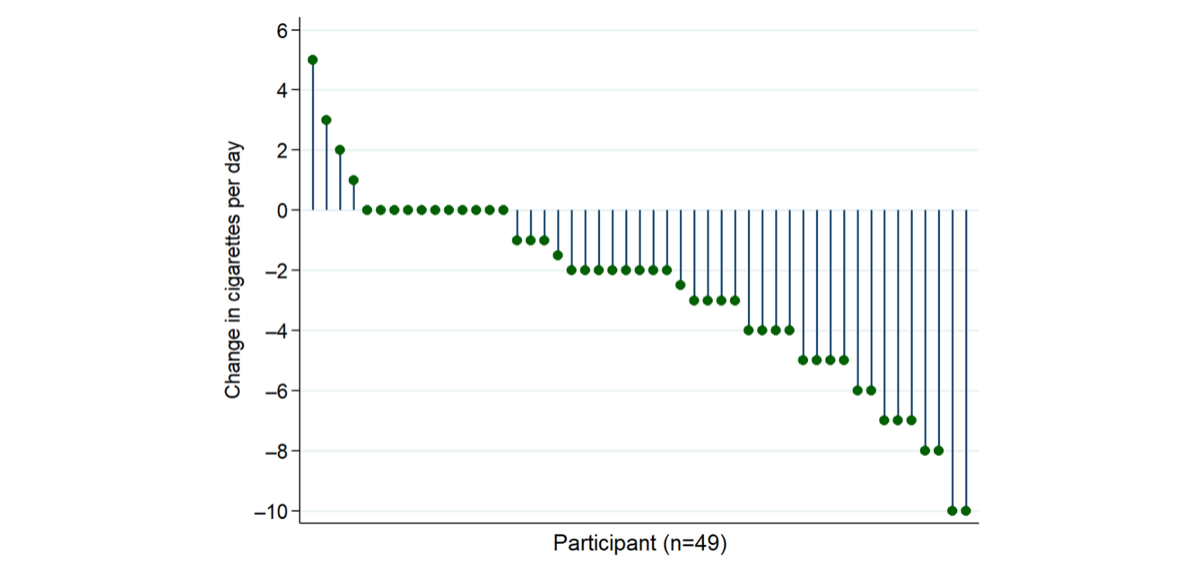
Change in cigarettes per day between baseline and follow-up.

### Changes in Measures Over the Course of the Study

Longitudinal trends in PA, smoking urges, and self-reported cigarette consumption between baseline and follow-up were examined to assess these behavior changes in the context of self-monitoring. After excluding outliers, participants’ mean daily urge increased by 0.10 (SD 0.82), whereas mean daily steps decreased by −82 (SD 1019); neither of these results was statistically significant. Self-reported number of cigarettes smoked per day significantly decreased from 12 (SD 8) at baseline to 9 (SD 8) at follow-up (Figure 5). Four participants quit smoking by the end of the study.

### Exit Survey Perceptions

Participants’ exit survey responses are summarized in Table 3. The majority of participants (68%) thought that PA influenced their smoking urges; among these, 76% believed that PA decreased their smoking urges. Fewer participants (37%) thought that higher smoking urges influenced their PA; among these, 61% believed that higher smoking urges decreased their PA. Just over half (59%) of participants thought the study helped them reduce smoking; among these, 79% reported decreases in cigarettes per day between enrollment and follow-up.

Finally, participants reported high levels of satisfaction with the study. Among exit survey respondents, the majority (67%) were extremely satisfied, and an additional 27% were moderately satisfied with the study.

**Table 3:** Results from follow-up online exit survey.

Characteristic		mActive-Smoke participants (N=49), n (%)
**IPAQ^a^**		
	Low	6 (12)
	Moderate	17 (35)
	High	26 (53)
**Cigarettes smoked per day**		
	0	4 (8)
	1-10	32 (65)
	>10	13 (27)
**During the past 3 months, have you tried to stop smoking?**		
	Yes	24 (49)
	No	25 (51)
**Does physical activity influence your smoking urges?**		
	Yes	33 (68)
	No	10 (20)
	Maybe	6 (12)
**How does physical activity influence your smoking urges?**		
	Increases the urge	8 (16)
	Decreases the urge	25 (51)
	No response	16 (33)
**Did wearing the Fitbit increase your awareness about daily physical activity?**		
	Yes	48 (98)
	No	1 (2)
**Do you think this study helped increase your daily physical activity?**		
	Yes	40 (82)
	No	3 (6)
	Maybe	6 (12)
**Did this study increase your awareness about smoking urges?**		
	Yes	44 (90)
	No	1 (2)
	Maybe	4 (8)
**Do you think this study helped you reduce smoking?**		
	Yes	29 (59)
	No	12 (25)
	Maybe	8 (16)

^a^IPAQ: International Physical Activity Questionnaire.

## Discussion

### Principal Findings

In the mActive-Smoke study, we found no day-level association between PA and smoking urges in the overall population, yet minute-level analyses of acute effects revealed modest inverse relationships between steps and urge for 30-, 60-, and 120-min time windows before urge reporting. There was also a small group of extreme responders who exhibited a more consistent and larger inverse relationship between steps and urge in both day- and minute-level analyses. Nevertheless, it is important to emphasize that analysis of extreme responders is highly exploratory and limited by a small sample size and not knowing times of prior smoking episodes.

### Comparison With Prior Work

This study was the first to leverage mHealth devices to evaluate the real-time association between PA and smoking urges in a longitudinal study of smokers in their natural environment. This study builds on prior literature leveraging digital technologies and EMA methods to collect real-world and real-time data on smoking behaviors [54-56]. Methodologically, this study sought to address limitations from prior studies exploring the relationship between smoking and PA under controlled conditions and with unreliable measurement tools. Regarding the former, a 2013 systematic review and meta-analysis concluded that short bouts of PA acutely decrease cigarette cravings by an average SMD of −2.03 between PA and control conditions [23]. Results from our 30-min level analysis were most comparable with those reported in this review, where PA interventions ranged in duration from 5 to 40 min. Despite the large effect size reported in this review, these results may be limited in generalizability because of study conditions. By conducting interventions in a controlled environment, behaviors observed in these studies were likely unrepresentative of those that might be observed in a real-world setting. Furthermore, these studies were limited to acute effects of PA on smoking urges, providing no evidence for how this interaction might play out in the long term. In the mActive-Smoke study, the use of Fitbit—an accurate and reliable wearable PA tracker [57]—allowed us to collect longitudinal PA data in participants’ natural settings in an effort to better capture real-world behaviors. Although the direction of our effect size was concordant with that reported in this review, its magnitude was far lower (approximately 0.2 reduction in urge on a 9-point Likert scale per 1000 steps). This discrepancy could be due, in part, to the lack of control over when the last cigarette was smoked before a bout of PA—a measure commonly implemented in prior experimental studies. Thus, it is possible that the decreased magnitude of association in the mActive-Smoke study resulted from a “flooring effect,” wherein a smoking episode in close proximity with urge reporting could significantly reduce the acute urge to smoke, leaving minimal room for subsequent changes in urge as a result of PA. On the other hand, the magnitude of our effect size may suggest that the effect of PA on urge may be less robust in a real-world, longitudinal setting.

Another batch of studies examined more longitudinal relationships between PA and smoking urges but relied on participant recall for data collection. For instance, Abrantes et al [27] conducted an exercise intervention study in which participants self-reported exercise in weekly activity logs. Prapavessis et al [26] also designed an exercise-intervention smoking cessation trial in which participants self-reported cigarette consumption on a weekly basis. As such, both studies were limited by subjective measurement tools, and their results were likely compromised by recall bias [31]—a major threat to the internal validity of studies using self-reported data [58,59]. In the mActive-Smoke study, use of Fitbit devices facilitated the objective collection of PA data, mitigating participant bias. Although smoking urges in our study were self-reported, the real-time nature of these measures likely rendered them less susceptible to recall bias compared with studies that relied on weeklong retrospective recall.

The design of the mActive-Smoke study allowed us to assess the association between PA and smoking behavior using approaches ranging from global to more granular. First, using a global approach, we analyzed daily steps and mean daily urge, allowing for a day-level comparison of these 2 measures. In addition to evaluating broad associations, secondary analyses were performed to explore more granular associations within hour and minute time intervals preceding urge reports, which allowed us to assess whether increased PA might acutely affect smoking urges. The presence of an inverse association between steps and urge in various granular time windows confirms findings from prior acute studies and extends them by suggesting that this effect may be particularly present in approximately 10% of individuals. Importantly, each of our analyses was performed in the participant’s natural environment, rather than a controlled research setting.

This study also highlights the potential benefits of integrating mHealth tools in the collection and assessment of behavioral data in translational research. Previous studies have suggested that mobile phone–based approaches are efficacious, user-friendly means for communicating with participants, providing instructions and modifying behaviors [60]. In addition to their practical utility, mHealth technology may also be leveraged to promote health equity. A 2016 survey by the Pew Research Center found that 92% of low-income adults own a cell phone and 64% own a smartphone [61]—a 14% increase since 2014 [35]. Among this demographic, one-fifth (21%) is smartphone dependent [61]—they rely heavily on their smartphones for Internet access and lack traditional broadband service in the home—and 63% report having used their smartphone to retrieve information about a health condition [35]. Thus, mHealth technology may provide a promising means of disseminating information and engaging members of these communities to make more health-conscious decisions [62].

Furthermore, the prevalence and frequency of mHealth use have been shown to be high among smokers, particularly those who are motivated to quit [63]. In the mActive-Smoke study, 98% of participants reported that wearing the Fitbit increased their awareness of daily PA, and 90% thought that the study increased their awareness about smoking urges. These findings strengthen the evidence behind self-monitoring and awareness on behavior change. Taylor et al [11] showed that the self-monitoring of smoking and PA behaviors led to reductions in smoking, which were determined to be independent of increases in PA [9]. In the mActive-Smoke study, no meaningful longitudinal changes were observed in PA or smoking urges, although daily cigarette consumption decreased by approximately 3 cigarettes per day between baseline and follow-up (*P*<.001). Given that this study was not focused on smoking reduction or cessation, these findings support the notion that greater self-awareness may lead to changes in smoking [9,11]. Taken together, this evidence suggests integration of mHealth devices in future smoking cessation and harm reduction trials in an effort to improve participant engagement and achieve desired behavioral outcomes.

Finally, this study offers provocative results when considering individual versus population-averaged effects that may inform precision medicine. Although population-averaged results were collectively null, 7 individuals—13% of our study population—showed a significant relationship between urge and steps in both day- and minute-level analyses. Among these extreme responders, 6 (11% of our study population) exhibited a consistently inverse association. Although these results are exploratory and hypothesis generating in nature, they suggest that PA could be targeted as a means to curb smoking urges among select individuals, an insight consistent with the PMI’s focus on individual variability in genes, environments, and lifestyles [64]. Further research is needed to determine whether equally robust inverse relationships between steps and urge could be replicated among these select individuals in an interventional study, which might help illuminate the underlying genetic, environmental, and lifestyle factors associated with these behaviors.

### Limitations

Although each participant contributed large quantities of individual data, the small number of participants in this pilot study may have limited our power to perform stratified analyses according to age, sex, and race. In addition, we did not collect data on daily cigarette consumption or on time since last cigarette because burdening participants with additional text messages may have reduced overall study adherence. These data, however, would have provided additional insight into the relationship between daily steps and mean daily urge by allowing us to assess an additional daily measure of smoking behavior. Furthermore, our failure to capture time since last cigarette may have resulted in a flooring effect, reducing our ability to measure the impact of PA on smoking urges because of the potential confounding effect of recent smoking episodes.

Although we were unable to control for time since last cigarette, we found a significant positive correlation between participants’ daily cigarette consumption in the exit survey and their mean urge over the course of the study, suggesting that these self-reported urges were valid indicators of smoking behavior. However, by allowing participants to report urges spontaneously and to dictate when they received the prompting messages, it is possible that some reporting bias was present.

In both primary and secondary analyses assessing the relationship between steps and urges, we did not control for intensity of exercise. In their 2014 meta-analysis [18], Haasova et al found that moderate and vigorous intensity exercise had the most benefits for reducing smoking cravings, although modest reductions were also reported for light exercise. Without controlling for exercise intensity in this study, the effect size seen in our secondary analyses may have been diluted by individuals with largely accumulated step counts made up of periods of low-intensity exercise. Although controlling for exercise intensity was outside the scope of this paper, we anticipate assessing its impact on urges in a future study.

For our primary and secondary analyses of steps versus urge, we chose to use FGLS because of the structure of data wherein the number of time points (T) exceeded the number of cross-sections (N). One limitation of this model was an inability to control for autocorrelation effects because of unbalanced panels in our dataset. Nonetheless, assessment of additional models, such as linear random effects, revealed no meaningful difference in effect size after controlling for autocorrelation. Another limitation is that FGLS, which originated in econometrics, has since been replaced by more modern methods in this field because it has been shown to produce inefficient estimates for data structures commonly seen in econometrics where N>T [51,65,66].

Finally, the sample of smokers in this study was very active, given that smokers tend to be considerably less active, on average, than the general population. Thus, it is possible that a ceiling effect was observed, wherein high levels of baseline PA limited participants’ abilities to further augment their PA during the study. Furthermore, our sample contained less heavy smokers compared with most prior studies, which generally used a minimum of 10 cigarettes per day as the threshold for study inclusion. Thus, results from this study ought to be interpreted with caution because of deviations in smoker demographics observed in our sample.

### Conclusions

Given the lack of a population-averaged longitudinal real-time association between PA and smoking urges, our results do not support broadly focusing resources on PA as a means to reduce smoking urges. Our data confirm results from prior studies, supporting the notion that acute bouts of PA can modestly curb smoking urges. This study also suggests that PA may significantly influence smoking urges among select individuals, an insight that aligns with the precision medicine model to focus on individual variability in health behaviors and outcomes. Furthermore, this study highlights the potential value of mHealth methods for assessing the interrelationships of cardiovascular health behaviors in the real world.

## Abbreviations

ASAQ: Arizona Smoking Assessment Questionnaire
CSV: comma separated values
EMA: ecological momentary assessment
FGLS: feasible generalized least squares
HR: heart rate
IPAQ: International Physical Activity Questionnaire
mHealth: mobile health
PA: physical activity
PMI: precision medicine initiative
SMD: standardized mean difference
SMS: short message service
